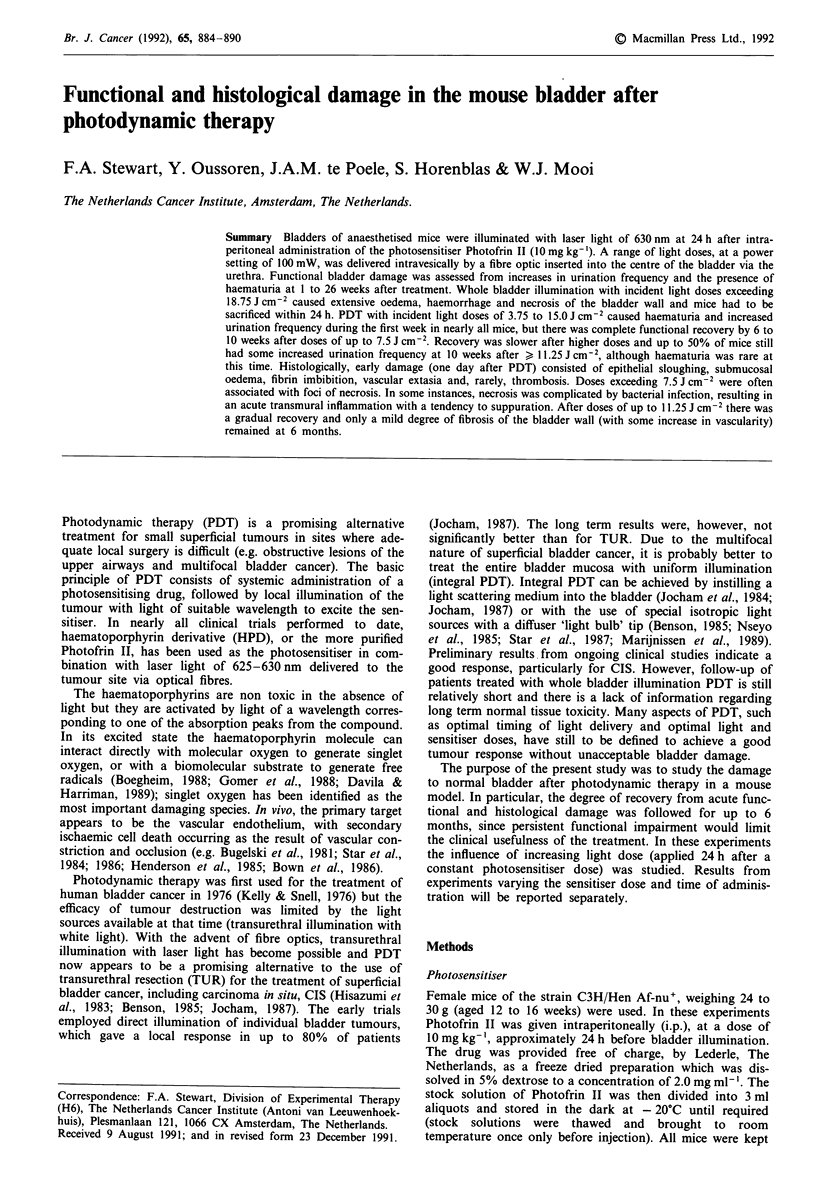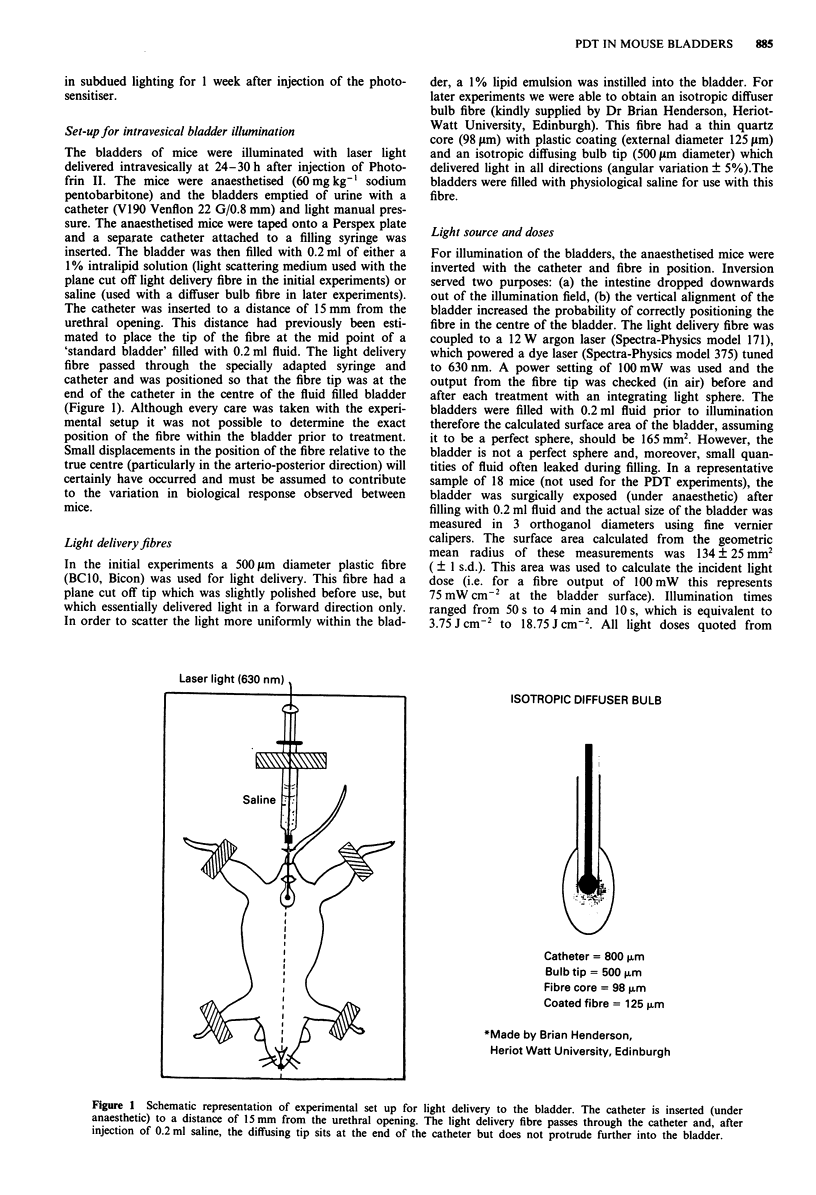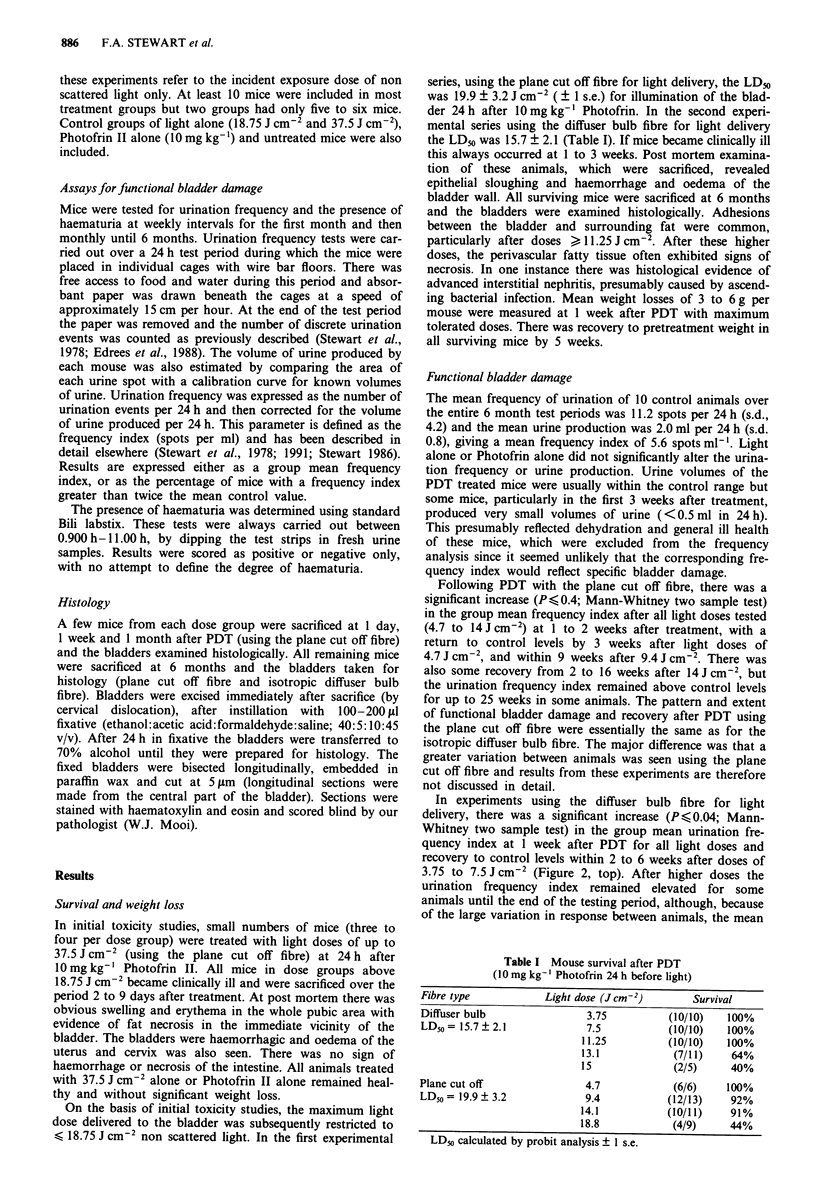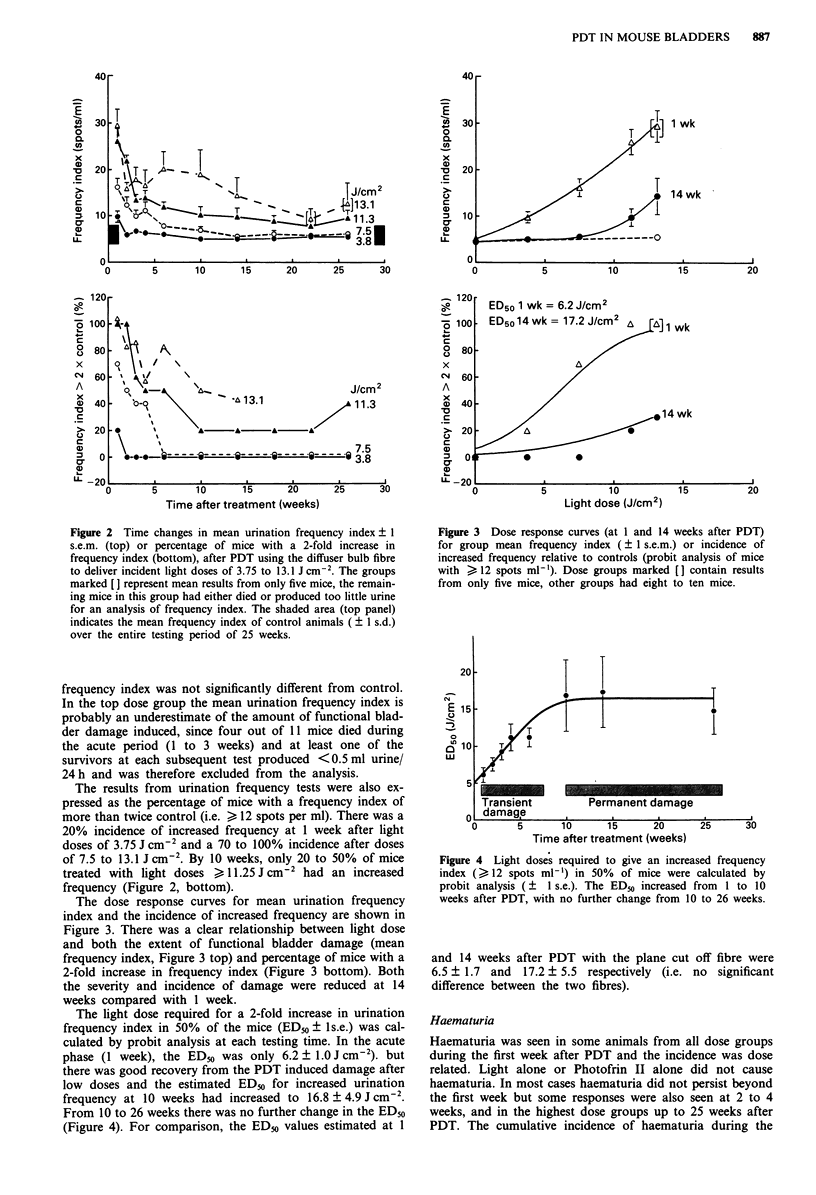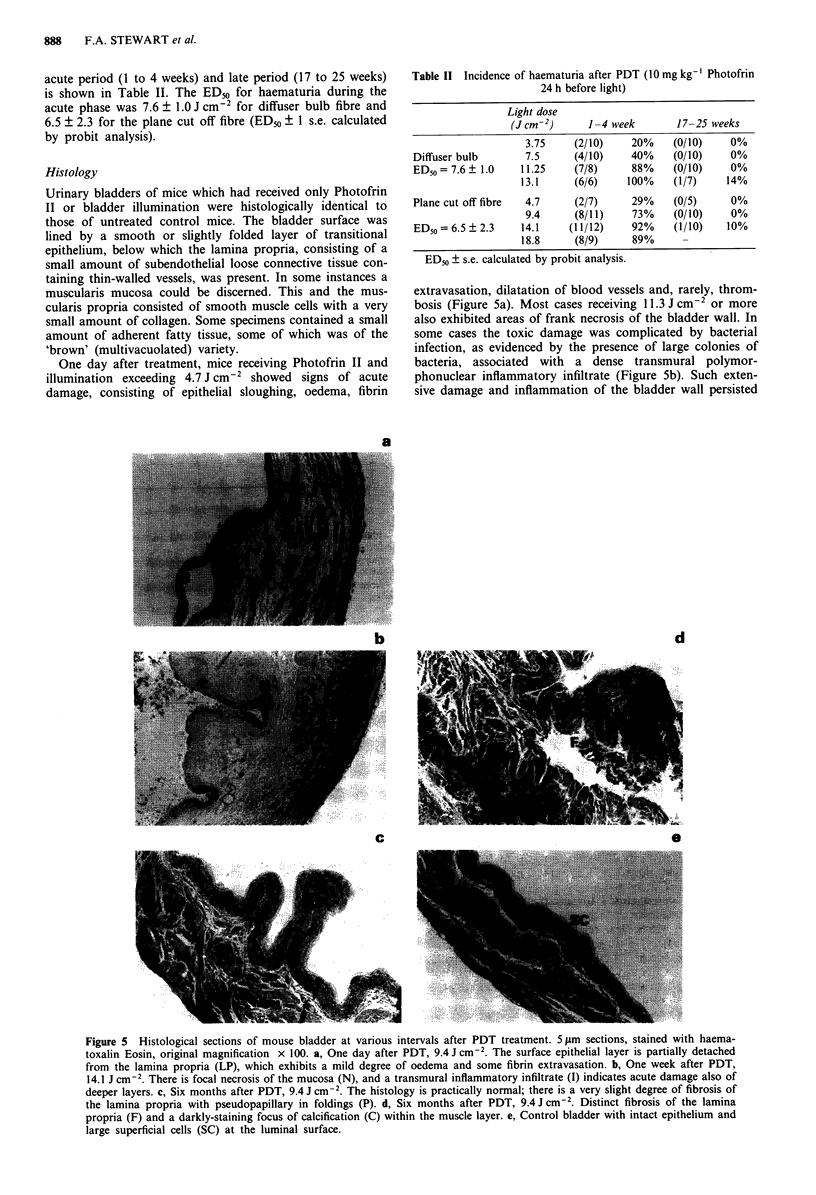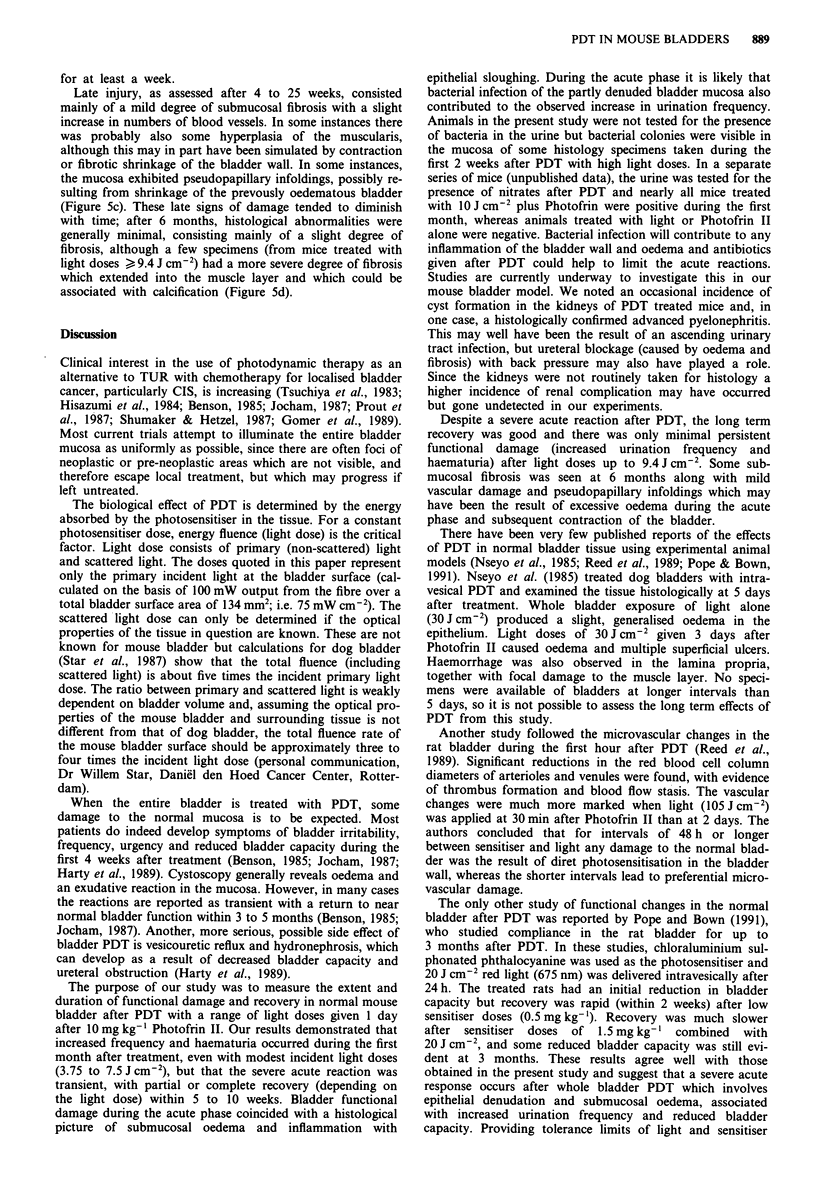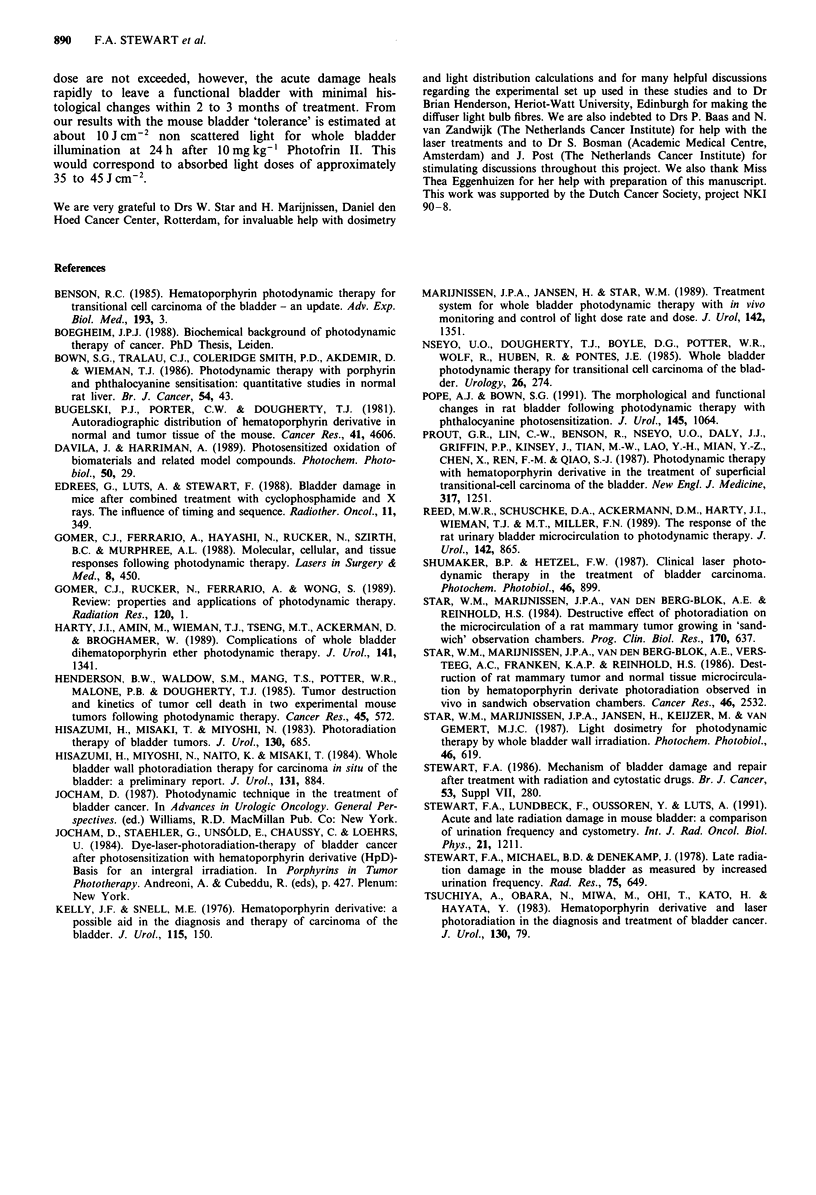# Functional and histological damage in the mouse bladder after photodynamic therapy.

**DOI:** 10.1038/bjc.1992.185

**Published:** 1992-06

**Authors:** F. A. Stewart, Y. Oussoren, J. A. te Poele, S. Horenblas, W. J. Mooi

**Affiliations:** The Netherlands Cancer Institute, Amsterdam.

## Abstract

**Images:**


Br. J. Cancer (1992), 65, 884-890 © Macmillan Press Ltd., 1992

### Functional and histological damage in the mouse bladder after photodynamic therapy

F.A. Stewart, Y. Oussoren, J.A.M. te Poele, S. Horenblas & W.J. Mooi

*The Netherlands Cancer Institute, Amsterdam, The Netherlands.*

**Summary** Bladders of anaesthetised mice were illuminated with laser light of 630 nm at 24 h after intraperitoneal administration of the photosensitiser Photofrin II (10 mg $kg^{-1}$). A range of light doses, at a power setting of 100 mW, was delivered intravesically by a fibre optic inserted into the centre of the bladder via the urethra. Functional bladder damage was assessed from increases in urination frequency and the presence of haematuria at 1 to 26 weeks after treatment. Whole bladder illumination with incident light doses exceeding 18.75 J $cm^{-2}$ caused extensive oedema, haemorrhage and necrosis of the bladder wall and mice had to be sacrificed within 24 h. PDT with incident light doses of 3.75 to 15.0 J $cm^{-2}$ caused haematuria and increased urination frequency during the first week in nearly all mice, but there was complete functional recovery by 6 to 10 weeks after doses of up to 7.5 J $cm^{-2}$. Recovery was slower after higher doses and up to 50% of mice still had some increased urination frequency at 10 weeks after $\geqslant$ 11.25 J $cm^{-2}$, although haematuria was rare at this time. Histologically, early damage (one day after PDT) consisted of epithelial sloughing, submucosal oedema, fibrin imbibition, vascular extasia and, rarely, thrombosis. Doses exceeding 7.5 J $cm^{-2}$ were often associated with foci of necrosis. In some instances, necrosis was complicated by bacterial infection, resulting in an acute transmural inflammation with a tendency to suppuration. After doses of up to 11.25 J $cm^{-2}$ there was a gradual recovery and only a mild degree of fibrosis of the bladder wall (with some increase in vascularity) remained at 6 months.

Photodynamic therapy (PDT) is a promising alternative treatment for small superficial tumours in sites where adequate local surgery is difficult (e.g. obstructive lesions of the upper airways and multifocal bladder cancer). The basic principle of PDT consists of systemic administration of a photosensitising drug, followed by local illumination of the tumour with light of suitable wavelength to excite the sensitiser. In nearly all clinical trials performed to date, haematoporphyrin derivative (HPD), or the more purified Photofrin II, has been used as the photosensitiser in combination with laser light of 625–630 nm delivered to the tumour site via optical fibres.

The haematoporphyrins are non toxic in the absence of light but they are activated by light of a wavelength corresponding to one of the absorption peaks from the compound. In its excited state the haematoporphyrin molecule can interact directly with molecular oxygen to generate singlet oxygen, or with a biomolecular substrate to generate free radicals (Boegheim, 1988; Gomer *et al.*, 1988; Davila & Harriman, 1989); singlet oxygen has been identified as the most important damaging species. *In vivo*, the primary target appears to be the vascular endothelium, with secondary ischaemic cell death occurring as the result of vascular constriction and occlusion (e.g. Bugelski *et al.*, 1981; Star *et al.*, 1984; 1986; Henderson *et al.*, 1985; Bown *et al.*, 1986).

Photodynamic therapy was first used for the treatment of human bladder cancer in 1976 (Kelly & Snell, 1976) but the efficacy of tumour destruction was limited by the light sources available at that time (transurethral illumination with white light). With the advent of fibre optics, transurethral illumination with laser light has become possible and PDT now appears to be a promising alternative to the use of transurethral resection (TUR) for the treatment of superficial bladder cancer, including carcinoma *in situ*, CIS (Hisazumi *et al.*, 1983; Benson, 1985; Jocham, 1987). The early trials employed direct illumination of individual bladder tumours, which gave a local response in up to 80% of patients (Jocham, 1987). The long term results were, however, not significantly better than for TUR. Due to the multifocal nature of superficial bladder cancer, it is probably better to treat the entire bladder mucosa with uniform illumination (integral PDT). Integral PDT can be achieved by instilling a light scattering medium into the bladder (Jocham *et al.*, 1984; Jocham, 1987) or with the use of special isotropic light sources with a diffuser 'light bulb' tip (Benson, 1985; Nseyo *et al.*, 1985; Star *et al.*, 1987; Marijnissen *et al.*, 1989). Preliminary results from ongoing clinical studies indicate a good response, particularly for CIS. However, follow-up of patients treated with whole bladder illumination PDT is still relatively short and there is a lack of information regarding long term normal tissue toxicity. Many aspects of PDT, such as optimal timing of light delivery and optimal light and sensitiser doses, have still to be defined to achieve a good tumour response without unacceptable bladder damage.

The purpose of the present study was to study the damage to normal bladder after photodynamic therapy in a mouse model. In particular, the degree of recovery from acute functional and histological damage was followed for up to 6 months, since persistent functional impairment would limit the clinical usefulness of the treatment. In these experiments the influence of increasing light dose (applied 24 h after a constant photosensitiser dose) was studied. Results from experiments varying the sensitiser dose and time of administration will be reported separately.

### Methods

### *Photosensitiser*

Female mice of the strain C3H/Hen Af-nu$^{+}$, weighing 24 to 30 g (aged 12 to 16 weeks) were used. In these experiments Photofrin II was given intraperitoneally (i.p.), at a dose of 10 mg $kg^{-1}$, approximately 24 h before bladder illumination. The drug was provided free of charge, by Lederle, The Netherlands, as a freeze dried preparation which was dissolved in 5% dextrose to a concentration of 2.0 mg $ml^{-1}$. The stock solution of Photofrin II was then divided into 3 ml aliquots and stored in the dark at $-20$°C until required (stock solutions were thawed and brought to room temperature once only before injection). All mice were kept

Correspondence: F.A. Stewart, Division of Experimental Therapy (H6), The Netherlands Cancer Institute (Antoni van Leeuwenhoekhuis), Plesmanlaan 121, 1066 CX Amsterdam, The Netherlands.
Received 9 August 1991; and in revised form 23 December 1991.

in subdued lighting for 1 week after injection of the photosensitiser.

*Set-up for intravesical bladder illumination*

The bladders of mice were illuminated with laser light delivered intravesically at 24–30 h after injection of Photofrin II. The mice were anaesthetised (60 mg kg$^{-1}$ sodium pentobarbitone) and the bladders emptied of urine with a catheter (V190 Venflon 22 G/0.8 mm) and light manual pressure. The anaesthetised mice were taped onto a Perspex plate and a separate catheter attached to a filling syringe was inserted. The bladder was then filled with 0.2 ml of either a 1% intralipid solution (light scattering medium used with the plane cut off light delivery fibre in the initial experiments) or saline (used with a diffuser bulb fibre in later experiments). The catheter was inserted to a distance of 15 mm from the urethral opening. This distance had previously been estimated to place the tip of the fibre at the mid point of a 'standard bladder' filled with 0.2 ml fluid. The light delivery fibre passed through the specially adapted syringe and catheter and was positioned so that the fibre tip was at the end of the catheter in the centre of the fluid filled bladder (Figure 1). Although every care was taken with the experimental setup it was not possible to determine the exact position of the fibre within the bladder prior to treatment. Small displacements in the position of the fibre relative to the true centre (particularly in the arterio-posterior direction) will certainly have occurred and must be assumed to contribute to the variation in biological response observed between mice.

*Light delivery fibres*

In the initial experiments a 500 μm diameter plastic fibre (BC10, Bicon) was used for light delivery. This fibre had a plane cut off tip which was slightly polished before use, but which essentially delivered light in a forward direction only. In order to scatter the light more uniformly within the bladder, a 1% lipid emulsion was instilled into the bladder. For later experiments we were able to obtain an isotropic diffuser bulb fibre (kindly supplied by Dr Brian Henderson, Heriot-Watt University, Edinburgh). This fibre had a thin quartz core (98 μm) with plastic coating (external diameter 125 μm) and an isotropic diffusing bulb tip (500 μm diameter) which delivered light in all directions (angular variation ± 5%).The bladders were filled with physiological saline for use with this fibre.

*Light source and doses*

For illumination of the bladders, the anaesthetised mice were inverted with the catheter and fibre in position. Inversion served two purposes: (a) the intestine dropped downwards out of the illumination field, (b) the vertical alignment of the bladder increased the probability of correctly positioning the fibre in the centre of the bladder. The light delivery fibre was coupled to a 12 W argon laser (Spectra-Physics model 171), which powered a dye laser (Spectra-Physics model 375) tuned to 630 nm. A power setting of 100 mW was used and the output from the fibre tip was checked (in air) before and after each treatment with an integrating light sphere. The bladders were filled with 0.2 ml fluid prior to illumination therefore the calculated surface area of the bladder, assuming it to be a perfect sphere, should be 165 mm$^2$. However, the bladder is not a perfect sphere and, moreover, small quantities of fluid often leaked during filling. In a representative sample of 18 mice (not used for the PDT experiments), the bladder was surgically exposed (under anaesthetic) after filling with 0.2 ml fluid and the actual size of the bladder was measured in 3 orthoganol diameters using fine vernier calipers. The surface area calculated from the geometric mean radius of these measurements was 134 ± 25 mm$^2$ ( ± 1 s.d.). This area was used to calculate the incident light dose (i.e. for a fibre output of 100 mW this represents 75 mW cm$^{-2}$ at the bladder surface). Illumination times ranged from 50 s to 4 min and 10 s, which is equivalent to 3.75 J cm$^{-2}$ to 18.75 J cm$^{-2}$. All light doses quoted from

**Figure 1** Schematic representation of experimental set up for light delivery to the bladder. The catheter is inserted (under anaesthetic) to a distance of 15 mm from the urethral opening. The light delivery fibre passes through the catheter and, after injection of 0.2 ml saline, the diffusing tip sits at the end of the catheter but does not protrude further into the bladder.

these experiments refer to the incident exposure dose of non scattered light only. At least 10 mice were included in most treatment groups but two groups had only five to six mice. Control groups of light alone (18.75 J $cm^{-2}$ and 37.5 J $cm^{-2}$), Photofrin II alone (10 mg $kg^{-1}$) and untreated mice were also included.

*Assays for functional bladder damage*

Mice were tested for urination frequency and the presence of haematuria at weekly intervals for the first month and then monthly until 6 months. Urination frequency tests were carried out over a 24 h test period during which the mice were placed in individual cages with wire bar floors. There was free access to food and water during this period and absorbant paper was drawn beneath the cages at a speed of approximately 15 cm per hour. At the end of the test period the paper was removed and the number of discrete urination events was counted as previously described (Stewart *et al.*, 1978; Edrees *et al.*, 1988). The volume of urine produced by each mouse was also estimated by comparing the area of each urine spot with a calibration curve for known volumes of urine. Urination frequency was expressed as the number of urination events per 24 h and then corrected for the volume of urine produced per 24 h. This parameter is defined as the frequency index (spots per ml) and has been described in detail elsewhere (Stewart *et al.*, 1978; 1991; Stewart 1986). Results are expressed either as a group mean frequency index, or as the percentage of mice with a frequency index greater than twice the mean control value.

The presence of haematuria was determined using standard Bili labstix. These tests were always carried out between 0.900 h–11.00 h, by dipping the test strips in fresh urine samples. Results were scored as positive or negative only, with no attempt to define the degree of haematuria.

*Histology*

A few mice from each dose group were sacrificed at 1 day, 1 week and 1 month after PDT (using the plane cut off fibre) and the bladders examined histologically. All remaining mice were sacrificed at 6 months and the bladders taken for histology (plane cut off fibre and isotropic diffuser bulb fibre). Bladders were excised immediately after sacrifice (by cervical dislocation), after instillation with 100–200 µl fixative (ethanol:acetic acid:formaldehyde:saline; 40:5:10:45 v/v). After 24 h in fixative the bladders were transferred to 70% alcohol until they were prepared for histology. The fixed bladders were bisected longitudinally, embedded in paraffin wax and cut at 5 µm (longitudinal sections were made from the central part of the bladder). Sections were stained with haematoxylin and eosin and scored blind by our pathologist (W.J. Mooi).

### Results

*Survival and weight loss*

In initial toxicity studies, small numbers of mice (three to four per dose group) were treated with light doses of up to 37.5 J $cm^{-2}$ (using the plane cut off fibre) at 24 h after 10 mg $kg^{-1}$ Photofrin II. All mice in dose groups above 18.75 J $cm^{-2}$ became clinically ill and were sacrificed over the period 2 to 9 days after treatment. At post mortem there was obvious swelling and erythema in the whole pubic area with evidence of fat necrosis in the immediate vicinity of the bladder. The bladders were haemorrhagic and oedema of the uterus and cervix was also seen. There was no sign of haemorrhage or necrosis of the intestine. All animals treated with 37.5 J $cm^{-2}$ alone or Photofrin II alone remained healthy and without significant weight loss.

On the basis of initial toxicity studies, the maximum light dose delivered to the bladder was subsequently restricted to $\leqslant$ 18.75 J $cm^{-2}$ non scattered light. In the first experimental series, using the plane cut off fibre for light delivery, the $LD_{50}$ was 19.9 ± 3.2 J $cm^{-2}$ (± 1 s.e.) for illumination of the bladder 24 h after 10 mg $kg^{-1}$ Photofrin. In the second experimental series using the diffuser bulb fibre for light delivery the $LD_{50}$ was 15.7 ± 2.1 (Table I). If mice became clinically ill this always occurred at 1 to 3 weeks. Post mortem examination of these animals, which were sacrificed, revealed epithelial sloughing and haemorrhage and oedema of the bladder wall. All surviving mice were sacrificed at 6 months and the bladders were examined histologically. Adhesions between the bladder and surrounding fat were common, particularly after doses $\geqslant$11.25 J $cm^{-2}$. After these higher doses, the perivascular fatty tissue often exhibited signs of necrosis. In one instance there was histological evidence of advanced interstitial nephritis, presumably caused by ascending bacterial infection. Mean weight losses of 3 to 6 g per mouse were measured at 1 week after PDT with maximum tolerated doses. There was recovery to pretreatment weight in all surviving mice by 5 weeks.

*Functional bladder damage*

The mean frequency of urination of 10 control animals over the entire 6 month test periods was 11.2 spots per 24 h (s.d., 4.2) and the mean urine production was 2.0 ml per 24 h (s.d. 0.8), giving a mean frequency index of 5.6 spots $ml^{-1}$. Light alone or Photofrin alone did not significantly alter the urination frequency or urine production. Urine volumes of the PDT treated mice were usually within the control range but some mice, particularly in the first 3 weeks after treatment, produced very small volumes of urine (<0.5 ml in 24 h). This presumably reflected dehydration and general ill health of these mice, which were excluded from the frequency analysis since it seemed unlikely that the corresponding frequency index would reflect specific bladder damage.

Following PDT with the plane cut off fibre, there was a significant increase ($P\leqslant 0.4$; Mann-Whitney two sample test) in the group mean frequency index after all light doses tested (4.7 to 14 J $cm^{-2}$) at 1 to 2 weeks after treatment, with a return to control levels by 3 weeks after light doses of 4.7 J $cm^{-2}$, and within 9 weeks after 9.4 J $cm^{-2}$. There was also some recovery from 2 to 16 weeks after 14 J $cm^{-2}$, but the urination frequency index remained above control levels for up to 25 weeks in some animals. The pattern and extent of functional bladder damage and recovery after PDT using the plane cut off fibre were essentially the same as for the isotropic diffuser bulb fibre. The major difference was that a greater variation between animals was seen using the plane cut off fibre and results from these experiments are therefore not discussed in detail.

In experiments using the diffuser bulb fibre for light delivery, there was a significant increase ($P\leqslant 0.04$; Mann-Whitney two sample test) in the group mean urination frequency index at 1 week after PDT for all light doses and recovery to control levels within 2 to 6 weeks after doses of 3.75 to 7.5 J $cm^{-2}$ (Figure 2, top). After higher doses the urination frequency index remained elevated for some animals until the end of the testing period, although, because of the large variation in response between animals, the mean

**Table I** Mouse survival after PDT (10 mg $kg^{-1}$ Photofrin 24 h before light)

| *Fibre type* | *Light dose (J $cm^{-2}$)* | *Survival* | |
|---|---|---|---|
| Diffuser bulb | 3.75 | (10/10) | 100% |
| $LD_{50}$ = 15.7 ± 2.1 | 7.5 | (10/10) | 100% |
| | 11.25 | (10/10) | 100% |
| | 13.1 | (7/11) | 64% |
| | 15 | (2/5) | 40% |
| Plane cut off | 4.7 | (6/6) | 100% |
| $LD_{50}$ = 19.9 ± 3.2 | 9.4 | (12/13) | 92% |
| | 14.1 | (10/11) | 91% |
| | 18.8 | (4/9) | 44% |

$LD_{50}$ calculated by probit analysis ± 1 s.e.

Figure 2 Time changes in mean urination frequency index ± 1 s.e.m. (top) or percentage of mice with a 2-fold increase in frequency index (bottom), after PDT using the diffuser bulb fibre to deliver incident light doses of 3.75 to 13.1 J cm$^{-2}$. The groups marked [ ] represent mean results from only five mice, the remaining mice in this group had either died or produced too little urine for an analysis of frequency index. The shaded area (top panel) indicates the mean frequency index of control animals ( ± 1 s.d.) over the entire testing period of 25 weeks.

Figure 3 Dose response curves (at 1 and 14 weeks after PDT) for group mean frequency index ( ± 1 s.e.m.) or incidence of increased frequency relative to controls (probit analysis of mice with ⩾12 spots ml$^{-1}$). Dose groups marked [ ] contain results from only five mice, other groups had eight to ten mice.

frequency index was not significantly different from control. In the top dose group the mean urination frequency index is probably an underestimate of the amount of functional bladder damage induced, since four out of 11 mice died during the acute period (1 to 3 weeks) and at least one of the survivors at each subsequent test produced <0.5 ml urine/24 h and was therefore excluded from the analysis.

The results from urination frequency tests were also expressed as the percentage of mice with a frequency index of more than twice control (i.e. ⩾12 spots per ml). There was a 20% incidence of increased frequency at 1 week after light doses of 3.75 J cm$^{-2}$ and a 70 to 100% incidence after doses of 7.5 to 13.1 J cm$^{-2}$. By 10 weeks, only 20 to 50% of mice treated with light doses ⩾11.25 J cm$^{-2}$ had an increased frequency (Figure 2, bottom).

The dose response curves for mean urination frequency index and the incidence of increased frequency are shown in Figure 3. There was a clear relationship between light dose and both the extent of functional bladder damage (mean frequency index, Figure 3 top) and percentage of mice with a 2-fold increase in frequency index (Figure 3 bottom). Both the severity and incidence of damage were reduced at 14 weeks compared with 1 week.

The light dose required for a 2-fold increase in urination frequency index in 50% of the mice ($ED_{50}$ ± 1s.e.) was calculated by probit analysis at each testing time. In the acute phase (1 week), the $ED_{50}$ was only 6.2 ± 1.0 J cm$^{-2}$). but there was good recovery from the PDT induced damage after low doses and the estimated $ED_{50}$ for increased urination frequency at 10 weeks had increased to 16.8 ± 4.9 J cm$^{-2}$. From 10 to 26 weeks there was no further change in the $ED_{50}$ (Figure 4). For comparison, the $ED_{50}$ values estimated at 1 and 14 weeks after PDT with the plane cut off fibre were 6.5 ± 1.7 and 17.2 ± 5.5 respectively (i.e. no significant difference between the two fibres).

Figure 4 Light doses required to give an increased frequency index (⩾12 spots ml$^{-1}$) in 50% of mice were calculated by probit analysis ( ± 1 s.e.). The $ED_{50}$ increased from 1 to 10 weeks after PDT, with no further change from 10 to 26 weeks.

*Haematuria*

Haematuria was seen in some animals from all dose groups during the first week after PDT and the incidence was dose related. Light alone or Photofrin II alone did not cause haematuria. In most cases haematuria did not persist beyond the first week but some responses were also seen at 2 to 4 weeks, and in the highest dose groups up to 25 weeks after PDT. The cumulative incidence of haematuria during the acute period (1 to 4 weeks) and late period (17 to 25 weeks) is shown in Table II. The $ED_{50}$ for haematuria during the acute phase was $7.6 \pm 1.0$ J cm$^{-2}$ for diffuser bulb fibre and $6.5 \pm 2.3$ for the plane cut off fibre ($ED_{50} \pm 1$ s.e. calculated by probit analysis).

*Histology*

Urinary bladders of mice which had received only Photofrin II or bladder illumination were histologically identical to those of untreated control mice. The bladder surface was lined by a smooth or slightly folded layer of transitional epithelium, below which the lamina propria, consisting of a small amount of subendothelial loose connective tissue containing thin-walled vessels, was present. In some instances a muscularis mucosa could be discerned. This and the muscularis propria consisted of smooth muscle cells with a very small amount of collagen. Some specimens contained a small amount of adherent fatty tissue, some of which was of the 'brown' (multivacuolated) variety.

One day after treatment, mice receiving Photofrin II and illumination exceeding 4.7 J cm$^{-2}$ showed signs of acute damage, consisting of epithelial sloughing, oedema, fibrin extravasation, dilatation of blood vessels and, rarely, thrombosis (Figure 5a). Most cases receiving 11.3 J cm$^{-2}$ or more also exhibited areas of frank necrosis of the bladder wall. In some cases the toxic damage was complicated by bacterial infection, as evidenced by the presence of large colonies of bacteria, associated with a dense transmural polymorphonuclear inflammatory infiltrate (Figure 5b). Such extensive damage and inflammation of the bladder wall persisted

**Table II** Incidence of haematuria after PDT (10 mg kg$^{-1}$ Photofrin 24 h before light)

| | *Light dose (J cm$^{-2}$)* | *1–4 week* | | *17–25 weeks* | |
|---|---|---|---|---|---|
| | 3.75 | (2/10) | 20% | (0/10) | 0% |
| Diffuser bulb | 7.5 | (4/10) | 40% | (0/10) | 0% |
| $ED_{50} = 7.6 \pm 1.0$ | 11.25 | (7/8) | 88% | (0/10) | 0% |
| | 13.1 | (6/6) | 100% | (1/7) | 14% |
| Plane cut off fibre | 4.7 | (2/7) | 29% | (0/5) | 0% |
| | 9.4 | (8/11) | 73% | (0/10) | 0% |
| $ED_{50} = 6.5 \pm 2.3$ | 14.1 | (11/12) | 92% | (1/10) | 10% |
| | 18.8 | (8/9) | 89% | – | |

$ED_{50} \pm$ s.e. calculated by probit analysis.

**Figure 5** Histological sections of mouse bladder at various intervals after PDT treatment. 5 µm sections, stained with haematoxalin Eosin, original magnification × 100. **a**, One day after PDT, 9.4 J cm$^{-2}$. The surface epithelial layer is partially detached from the lamina propria (LP), which exhibits a mild degree of oedema and some fibrin extravasation. **b**, One week after PDT, 14.1 J cm$^{-2}$. There is focal necrosis of the mucosa (N), and a transmural inflammatory infiltrate (I) indicates acute damage also of deeper layers. **c**, Six months after PDT, 9.4 J cm$^{-2}$. The histology is practically normal; there is a very slight degree of fibrosis of the lamina propria with pseudopapillary in foldings (P). **d**, Six months after PDT, 9.4 J cm$^{-2}$. Distinct fibrosis of the lamina propria (F) and a darkly-staining focus of calcification (C) within the muscle layer. **e**, Control bladder with intact epithelium and large superficial cells (SC) at the luminal surface.

for at least a week.

Late injury, as assessed after 4 to 25 weeks, consisted mainly of a mild degree of submucosal fibrosis with a slight increase in numbers of blood vessels. In some instances there was probably also some hyperplasia of the muscularis, although this may in part have been simulated by contraction or fibrotic shrinkage of the bladder wall. In some instances, the mucosa exhibited pseudopapillary infoldings, possibly resulting from shrinkage of the prevously oedematous bladder (Figure 5c). These late signs of damage tended to diminish with time; after 6 months, histological abnormalities were generally minimal, consisting mainly of a slight degree of fibrosis, although a few specimens (from mice treated with light doses $\geqslant 9.4\ \mathrm{J\ cm^{-2}}$) had a more severe degree of fibrosis which extended into the muscle layer and which could be associated with calcification (Figure 5d).

### Discussion

Clinical interest in the use of photodynamic therapy as an alternative to TUR with chemotherapy for localised bladder cancer, particularly CIS, is increasing (Tsuchiya *et al.*, 1983; Hisazumi *et al.*, 1984; Benson, 1985; Jocham, 1987; Prout *et al.*, 1987; Shumaker & Hetzel, 1987; Gomer *et al.*, 1989). Most current trials attempt to illuminate the entire bladder mucosa as uniformly as possible, since there are often foci of neoplastic or pre-neoplastic areas which are not visible, and therefore escape local treatment, but which may progress if left untreated.

The biological effect of PDT is determined by the energy absorbed by the photosensitiser in the tissue. For a constant photosensitiser dose, energy fluence (light dose) is the critical factor. Light dose consists of primary (non-scattered) light and scattered light. The doses quoted in this paper represent only the primary incident light at the bladder surface (calculated on the basis of 100 mW output from the fibre over a total bladder surface area of 134 $\mathrm{mm^2}$; i.e. 75 $\mathrm{mW\ cm^{-2}}$). The scattered light dose can only be determined if the optical properties of the tissue in question are known. These are not known for mouse bladder but calculations for dog bladder (Star *et al.*, 1987) show that the total fluence (including scattered light) is about five times the incident primary light dose. The ratio between primary and scattered light is weakly dependent on bladder volume and, assuming the optical properties of the mouse bladder and surrounding tissue is not different from that of dog bladder, the total fluence rate of the mouse bladder surface should be approximately three to four times the incident light dose (personal communication, Dr Willem Star, Daniël den Hoed Cancer Center, Rotterdam).

When the entire bladder is treated with PDT, some damage to the normal mucosa is to be expected. Most patients do indeed develop symptoms of bladder irritability, frequency, urgency and reduced bladder capacity during the first 4 weeks after treatment (Benson, 1985; Jocham, 1987; Harty *et al.*, 1989). Cystoscopy generally reveals oedema and an exudative reaction in the mucosa. However, in many cases the reactions are reported as transient with a return to near normal bladder function within 3 to 5 months (Benson, 1985; Jocham, 1987). Another, more serious, possible side effect of bladder PDT is vesicouretic reflux and hydronephrosis, which can develop as a result of decreased bladder capacity and ureteral obstruction (Harty *et al.*, 1989).

The purpose of our study was to measure the extent and duration of functional damage and recovery in normal mouse bladder after PDT with a range of light doses given 1 day after 10 $\mathrm{mg\ kg^{-1}}$ Photofrin II. Our results demonstrated that increased frequency and haematuria occurred during the first month after treatment, even with modest incident light doses (3.75 to 7.5 $\mathrm{J\ cm^{-2}}$), but that the severe acute reaction was transient, with partial or complete recovery (depending on the light dose) within 5 to 10 weeks. Bladder functional damage during the acute phase coincided with a histological picture of submucosal oedema and inflammation with epithelial sloughing. During the acute phase it is likely that bacterial infection of the partly denuded bladder mucosa also contributed to the observed increase in urination frequency. Animals in the present study were not tested for the presence of bacteria in the urine but bacterial colonies were visible in the mucosa of some histology specimens taken during the first 2 weeks after PDT with high light doses. In a separate series of mice (unpublished data), the urine was tested for the presence of nitrates after PDT and nearly all mice treated with 10 $\mathrm{J\ cm^{-2}}$ plus Photofrin were positive during the first month, whereas animals treated with light or Photofrin II alone were negative. Bacterial infection will contribute to any inflammation of the bladder wall and oedema and antibiotics given after PDT could help to limit the acute reactions. Studies are currently underway to investigate this in our mouse bladder model. We noted an occasional incidence of cyst formation in the kidneys of PDT treated mice and, in one case, a histologically confirmed advanced pyelonephritis. This may well have been the result of an ascending urinary tract infection, but ureteral blockage (caused by oedema and fibrosis) with back pressure may also have played a role. Since the kidneys were not routinely taken for histology a higher incidence of renal complication may have occurred but gone undetected in our experiments.

Despite a severe acute reaction after PDT, the long term recovery was good and there was only minimal persistent functional damage (increased urination frequency and haematuria) after light doses up to 9.4 $\mathrm{J\ cm^{-2}}$. Some submucosal fibrosis was seen at 6 months along with mild vascular damage and pseudopapillary infoldings which may have been the result of excessive oedema during the acute phase and subsequent contraction of the bladder.

There have been very few published reports of the effects of PDT in normal bladder tissue using experimental animal models (Nseyo *et al.*, 1985; Reed *et al.*, 1989; Pope & Bown, 1991). Nseyo *et al.* (1985) treated dog bladders with intravesical PDT and examined the tissue histologically at 5 days after treatment. Whole bladder exposure of light alone (30 $\mathrm{J\ cm^{-2}}$) produced a slight, generalised oedema in the epithelium. Light doses of 30 $\mathrm{J\ cm^{-2}}$ given 3 days after Photofrin II caused oedema and multiple superficial ulcers. Haemorrhage was also observed in the lamina propria, together with focal damage to the muscle layer. No specimens were available of bladders at longer intervals than 5 days, so it is not possible to assess the long term effects of PDT from this study.

Another study followed the microvascular changes in the rat bladder during the first hour after PDT (Reed *et al.*, 1989). Significant reductions in the red blood cell column diameters of arterioles and venules were found, with evidence of thrombus formation and blood flow stasis. The vascular changes were much more marked when light (105 $\mathrm{J\ cm^{-2}}$) was applied at 30 min after Photofrin II than at 2 days. The authors concluded that for intervals of 48 h or longer between sensitiser and light any damage to the normal bladder was the result of diret photosensitisation in the bladder wall, whereas the shorter intervals lead to preferential microvascular damage.

The only other study of functional changes in the normal bladder after PDT was reported by Pope and Bown (1991), who studied compliance in the rat bladder for up to 3 months after PDT. In these studies, chloraluminium sulphonated phthalocyanine was used as the photosensitiser and 20 $\mathrm{J\ cm^{-2}}$ red light (675 nm) was delivered intravesically after 24 h. The treated rats had an initial reduction in bladder capacity but recovery was rapid (within 2 weeks) after low sensitiser doses (0.5 $\mathrm{mg\ kg^{-1}}$). Recovery was much slower after sensitiser doses of 1.5 $\mathrm{mg\ kg^{-1}}$ combined with 20 $\mathrm{J\ cm^{-2}}$, and some reduced bladder capacity was still evident at 3 months. These results agree well with those obtained in the present study and suggest that a severe acute response occurs after whole bladder PDT which involves epithelial denudation and submucosal oedema, associated with increased urination frequency and reduced bladder capacity. Providing tolerance limits of light and sensitiser dose are not exceeded, however, the acute damage heals rapidly to leave a functional bladder with minimal histological changes within 2 to 3 months of treatment. From our results with the mouse bladder 'tolerance' is estimated at about $10\,J\,cm^{-2}$ non scattered light for whole bladder illumination at 24 h after $10\,mg\,kg^{-1}$ Photofrin II. This would correspond to absorbed light doses of approximately 35 to $45\,J\,cm^{-2}$.

We are very grateful to Drs W. Star and H. Marijnissen, Daniel den Hoed Cancer Center, Rotterdam, for invaluable help with dosimetry and light distribution calculations and for many helpful discussions regarding the experimental set up used in these studies and to Dr Brian Henderson, Heriot-Watt University, Edinburgh for making the diffuser light bulb fibres. We are also indebted to Drs P. Baas and N. van Zandwijk (The Netherlands Cancer Institute) for help with the laser treatments and to Dr S. Bosman (Academic Medical Centre, Amsterdam) and J. Post (The Netherlands Cancer Institute) for stimulating discussions throughout this project. We also thank Miss Thea Eggenhuizen for her help with preparation of this manuscript. This work was supported by the Dutch Cancer Society, project NKI 90-8.

### References

BENSON, R.C. (1985). Hematoporphyrin photodynamic therapy for transitional cell carcinoma of the bladder – an update. *Adv. Exp. Biol. Med.*, **193**, 3.

BOEGHEIM, J.P.J. (1988). Biochemical background of photodynamic therapy of cancer. PhD Thesis, Leiden.

BOWN, S.G., TRALAU, C.J., COLERIDGE SMITH, P.D., AKDEMIR, D. & WIEMAN, T.J. (1986). Photodynamic therapy with porphyrin and phthalocyanine sensitisation: quantitative studies in normal rat liver. *Br. J. Cancer*, **54**, 43.

BUGELSKI, P.J., PORTER, C.W. & DOUGHERTY, T.J. (1981). Autoradiographic distribution of hematoporphyrin derivative in normal and tumor tissue of the mouse. *Cancer Res.*, **41**, 4606.

DAVILA, J. & HARRIMAN, A. (1989). Photosensitized oxidation of biomaterials and related model compounds. *Photochem. Photobiol.*, **50**, 29.

EDREES, G., LUTS, A. & STEWART, F. (1988). Bladder damage in mice after combined treatment with cyclophosphamide and X rays. The influence of timing and sequence. *Radiother. Oncol.*, **11**, 349.

GOMER, C.J., FERRARIO, A., HAYASHI, N., RUCKER, N., SZIRTH, B.C. & MURPHREE, A.L. (1988). Molecular, cellular, and tissue responses following photodynamic therapy. *Lasers in Surgery & Med.*, **8**, 450.

GOMER, C.J., RUCKER, N., FERRARIO, A. & WONG, S. (1989). Review: properties and applications of photodynamic therapy. *Radiation Res.*, **120**, 1.

HARTY, J.I., AMIN, M., WIEMAN, T.J., TSENG, M.T., ACKERMAN, D. & BROGHAMER, W. (1989). Complications of whole bladder dihematoporphyrin ether photodynamic therapy. *J. Urol.*, **141**, 1341.

HENDERSON, B.W., WALDOW, S.M., MANG, T.S., POTTER, W.R., MALONE, P.B. & DOUGHERTY, T.J. (1985). Tumor destruction and kinetics of tumor cell death in two experimental mouse tumors following photodynamic therapy. *Cancer Res.*, **45**, 572.

HISAZUMI, H., MISAKI, T. & MIYOSHI, N. (1983). Photoradiation therapy of bladder tumors. *J. Urol.*, **130**, 685.

HISAZUMI, H., MIYOSHI, N., NAITO, K. & MISAKI, T. (1984). Whole bladder wall photoradiation therapy for carcinoma *in situ* of the bladder: a preliminary report. *J. Urol.*, **131**, 884.

JOCHAM, D. (1987). Photodynamic technique in the treatment of bladder cancer. In *Advances in Urologic Oncology. General Perspectives.* (ed.) Williams, R.D. MacMillan Pub. Co: New York.

JOCHAM, D., STAEHLER, G., UNSÖLD, E., CHAUSSY, C. & LOEHRS, U. (1984). Dye-laser-photoradiation-therapy of bladder cancer after photosensitization with hematoporphyrin derivative (HpD)-Basis for an intergral irradiation. In *Porphyrins in Tumor Phototherapy*. Andreoni, A. & Cubeddu, R. (eds), p. 427. Plenum: New York.

KELLY, J.F. & SNELL, M.E. (1976). Hematoporphyrin derivative: a possible aid in the diagnosis and therapy of carcinoma of the bladder. *J. Urol.*, **115**, 150.

MARIJNISSEN, J.P.A., JANSEN, H. & STAR, W.M. (1989). Treatment system for whole bladder photodynamic therapy with *in vivo* monitoring and control of light dose rate and dose. *J. Urol*, **142**, 1351.

NSEYO, U.O., DOUGHERTY, T.J., BOYLE, D.G., POTTER, W.R., WOLF, R., HUBEN, R. & PONTES, J.E. (1985). Whole bladder photodynamic therapy for transitional cell carcinoma of the bladder. *Urology*, **26**, 274.

POPE, A.J. & BOWN, S.G. (1991). The morphological and functional changes in rat bladder following photodynamic therapy with phthalocyanine photosensitization. *J. Urol.*, **145**, 1064.

PROUT, G.R., LIN, C.-W., BENSON, R., NSEYO, U.O., DALY, J.J., GRIFFIN, P.P., KINSEY, J., TIAN, M.-W., LAO, Y.-H., MIAN, Y.-Z., CHEN, X., REN, F.-M. & QIAO, S.-J. (1987). Photodynamic therapy with hematoporphyrin derivative in the treatment of superficial transitional-cell carcinoma of the bladder. *New Engl. J. Medicine*, **317**, 1251.

REED, M.W.R., SCHUSCHKE, D.A., ACKERMANN, D.M., HARTY, J.I., WIEMAN, T.J. & M.T., MILLER, F.N. (1989). The response of the rat urinary bladder microcirculation to photodynamic therapy. *J. Urol.*, **142**, 865.

SHUMAKER, B.P. & HETZEL, F.W. (1987). Clinical laser photodynamic therapy in the treatment of bladder carcinoma. *Photochem. Photobiol.*, **46**, 899.

STAR, W.M., MARIJNISSEN, J.P.A., VAN DEN BERG-BLOK, A.E. & REINHOLD, H.S. (1984). Destructive effect of photoradiation on the microcirculation of a rat mammary tumor growing in 'sandwich' observation chambers. *Prog. Clin. Biol. Res.*, **170**, 637.

STAR, W.M., MARIJNISSEN, J.P.A., VAN DEN BERG-BLOK, A.E., VERSTEEG, A.C., FRANKEN, K.A.P. & REINHOLD, H.S. (1986). Destruction of rat mammary tumor and normal tissue microcirculation by hematoporphyrin derivate photoradiation observed in vivo in sandwich observation chambers. *Cancer Res.*, **46**, 2532.

STAR, W.M., MARIJNISSEN, J.P.A., JANSEN, H., KEIJZER, M. & VAN GEMERT, M.J.C. (1987). Light dosimetry for photodynamic therapy by whole bladder wall irradiation. *Photochem. Photobiol.*, **46**, 619.

STEWART, F.A. (1986). Mechanism of bladder damage and repair after treatment with radiation and cytostatic drugs. *Br. J. Cancer*, **53**, Suppl VII, 280.

STEWART, F.A., LUNDBECK, F., OUSSOREN, Y. & LUTS, A. (1991). Acute and late radiation damage in mouse bladder: a comparison of urination frequency and cystometry. *Int. J. Rad. Oncol. Biol. Phys.*, **21**, 1211.

STEWART, F.A., MICHAEL, B.D. & DENEKAMP, J. (1978). Late radiation damage in the mouse bladder as measured by increased urination frequency. *Rad. Res.*, **75**, 649.

TSUCHIYA, A., OBARA, N., MIWA, M., OHI, T., KATO, H. & HAYATA, Y. (1983). Hematoporphyrin derivative and laser photoradiation in the diagnosis and treatment of bladder cancer. *J. Urol.*, **130**, 79.